# Golf and Physical Health: A Systematic Review

**DOI:** 10.1007/s40279-022-01732-w

**Published:** 2022-08-06

**Authors:** Graeme G. Sorbie, Alexander J. Beaumont, Ashley K. Williams, David Lavallee

**Affiliations:** 1 Division of Sport and Exercise Sciences, School of Applied Sciences, Abertay University, Dundee, Scotland, UK; 2 School of Science, Technology and Health, York St John University, York, UK

## Abstract

**Background:**

No previous systematic review has examined the physical health benefits of playing golf or caddying.

**Objective:**

To establish the influence of golf participation and physical health in golfers and caddies. More specifically, the review intended to explore the domains of cardiovascular, metabolic and musculoskeletal health, in addition to body composition.

**Design:**

Systematic review.

**Data Sources:**

Electronic literature searches were conducted using PubMed, SPORTDiscus and CINAHL databases in July 2021.

**Eligibility Criteria:**

Experimental (randomised controlled trials, quasi-experiment, pre-post) and non-experimental (case–control, cross-sectional, cohort) articles relating to health and golf, written in English and published in peer-reviewed journals.

**Results:**

Of the 572 articles initially identified, 109 full-text articles were assessed for eligibility with 23 meeting the inclusion criteria. Sixteen articles were rated ‘good 'and seven ‘fair’. The influence of golf on physical health was mixed, although various articles displayed improvements in balance, systolic blood pressure (SBP) and diastolic blood pressure (DBP), high density lipoprotein-cholesterol (HDL-C) and the ratio of HDL to total cholesterol within golfers. Caddies observed improvements in bone mineral density (BMD), stiffness index and strength. Most of the findings indicate that playing golf or caddying does not influence body mass index (BMI); however, playing golf can positively change other body composition markers such as lean and fat mass.

**Conclusion:**

This review demonstrated that golf participation may be an effective method for improving musculoskeletal and cardiovascular health, although mixed findings were observed. Moreover, limited longitudinal evidence suggests that playing golf can positively impact metabolic health and the influence on body composition may be parameter dependent. Additionally, the initial evidence suggests that caddying may improve musculoskeletal health. However, the studies included were limited by their methodological inconsistencies such as: study design, participant demographics and intervention prescription.

**PROSPERO Registration:**

CRD42021267664.

## Key Points

**Table:** 

This is the first systematic review of literature having investigated the influence of golf and caddying on physical health.
Golf may be beneficial for musculoskeletal, metabolic and cardiovascular health; however, findings were mixed and likely dependent on the parameters assessed, in addition to golfer’s age, baseline health status and the golf activity performed. Caddying may be effective for improvements and maintenance of lower limb muscular strength.
Future research should consider the intervention type and duration to understand the long-term impact of golf on physical health.

## Introduction

The popularity of golf is ever-growing, with a 2.3 million increase in on-course participation in Great Britain and Ireland in 2020 [1], with 2.2% of the population reported to play golf bi-weekly [2]. More females are participating in golf [1], which is also a game accessible to people of all ages, from the young to the old. Beyond golf participation, caddies contribute to the sport at all levels of the game, from recreational to professional. Typically, the caddie’s roles include, but are not limited to, carrying golf bags, attending to the flag stick, and providing strategic support to the golfer [3–6].

The average age of registered golfers within the UK is 41 years [1], with a forecasted 42% of golfers within Europe being older than 60 years [7]. The World Health Organisation (WHO) [8] predicts that in less than a decade, one in six people will be older than 60 years. Whilst ageing is not a direct cause for cardiovascular diseases (CVD) [9], it is associated with biological degeneration and senescence, which heightens the risk for disease and health complications [10]. In addition, falls are more common with advancing age and with an increase in frailty [11]. Furthermore, developing musculoskeletal disorders within older adults are also rising and are amongst the most common health issues [12, 13]. Within older adults, musculoskeletal disorders such as rheumatoid arthritis and osteoarthritis are associated with increased risk of falls [13, 14]. While the population is living longer, these extra years may not be spent in good health [8], thus limiting the health span—the phase of life without disability and free from serious illness [15]. A loss of physiological function attenuates functional status and heightens the risk of mortality and morbidity [16]. In turn, preservation of physiological function is paramount in the attainment of optimal longevity, health span [15] and in delaying future age-related chronic diseases. Therefore, effective lifestyle-behavioural strategies, such as physical activity (PA), have been considered as primary approaches in the attempt to slow the declines in physical health and functional independence, in order to increase health span [16].

Physical activity is a cost-effective, non-pharmacological method for improving health, supported by a curvilinear dose–response relationship between PA level and health benefits [17]. Golf presents an opportunity to increase PA and provides improvements in risk factors for CVD and metabolic and musculoskeletal health [18]. The metabolic cost and heart-rate responses through playing golf are dependent on numerous factors. Examples include riding an electric cart compared to carrying clubs or using a pull cart [19], carrying a bag with one or two straps [20, 21], and playing on a hilly course, although this is debated between studies [19, 22]. Additionally, caddies can be expected to carry a bag of at least 12.5 kg [3], which presents an additional physical challenge to walking alone, during an on-course round of golf. General golf is considered to be a moderate-intensity activity with a metabolic doequivalent (METs) of 4.8 [18, 23], with reports that a nine-hole golf round elicits 46% of peak MET for a healthy older (64 ± 8 years) population [24]. The relative intensity of activity varies depending on health status [24] and also increases progressively with age [25]. A systematic review identified that while a single round was sufficient to achieve the American College of Sports Medicine recommendations for energy expenditure, the mode of club transportation, age and skill level of golfers all contribute to variations in PA level [26].

A scoping review was recently conducted to establish the existing body of literature related to golf and health [18]. A variety of categories were explored concerning both physical and mental health, with a recommendation for a systematic review to be conducted to further the understanding between golf and health. Moreover, the focus has long been placed on golfers, while the effect of golf caddying on health has started to receive attention [3]. Given the absence of a focused systematic review on physical health and golf derived from peer-reviewed academic literature, the present systematic review aimed to establish the influence of golf participation and physical health in golfers and caddies. More specifically, we intended to explore the domains of cardiovascular, metabolic and musculoskeletal health, in addition to body composition.

## Methods

This systematic literature review was conducted in accordance with the recommendations for the Preferred Reporting Items for Systematic Review and Meta-Analysis (PRISMA) statement [27]. The systematic review was registered using the PROSPERO International database of systematic review protocols (Registration Number: CRD42021267664).

### Search Terms and Criteria for Inclusion

An electronic literature search was conducted using PubMed, SPORTDiscus and CINAHL databases in July 2021, to identify available research studies that were related to golf and health. In all databases, a title and abstract search was conducted using a string of search terms that included: (Golf* OR Caddy OR Caddie OR Caddies OR Caddying) AND (Health OR Physical OR Cardiovascular OR Musculoskeletal OR MSK OR MSD OR Metabolic). Additional filters were applied to include studies only published in English. Primary source, peer-reviewed articles were eligible to be included if they contained data relating to golf and health. Specific inclusion criteria were as follows:

- Peer-reviewed articles.
- Primary research articles with any study designs (i.e., observational, cross-sectional, experimental studies) that presented a non-golf control group (cross-sectional) or determined changes (cohort or experimental) with statistical analyses.
- Golfers of any age and skill level, including both competitive and recreational golfers.
- Caddies of any age and level.
- All forms of on-course and off-course golf (18 holes, nine holes, short-form golf, driving ranges, etc.).
- English language studies.
- Assessed markers of physical health pertaining to the domains of cardiovascular, metabolic, musculoskeletal health and body composition.

Conference proceedings, reviews, clinical commentaries, case reports, theses and dissertations were excluded. Titles and abstracts were reviewed independently by two authors (AJB and GGS) for relevance, followed by full-text screening to assess eligibility based on the inclusion and exclusion criteria. Reference lists of relevant reviews were screened for any additional articles that may have been missed from the electronic database search (AJB and GGS). During the review process, a third author (AKW) arbitrated any uncertainties in study inclusion.

### Data Extraction

Included articles and relevant data were extracted into a custom Microsoft Excel sheet (Version 2016) by two authors independently (AJB and GGS). Extracted data included: (1) study details, such as authors, date of publication and study design; (2) participant characteristics, such as golf status (i.e., golfer or caddie), age, sex and skill level; (3) cardiovascular measurements including systolic (SBP) and diastolic blood pressure (DBP), cardiac function, inflammatory blood markers, maximum oxygen uptake ($\dot{V}{\text{O}}_{2\max }$) and associations with CVD risk; (4) musculoskeletal variables including balance, flexibility, muscle mass, thickness, strength and endurance, bone mineral density (BMD), bone mineral content (BMC) and physical competency measures; (5) metabolic variables including blood lipid profiles, bone metabolic markers and bone resorption rates; (6) body composition measurements including body mass index (BMI), fat mass, abdominal skinfold thickness and waist circumference.

### Data Analysis and Quality Assessment

Narrative data analysis was conducted in each of the four domains described previously. The National Heart, Lung, and Blood Institute (NHLBI) risk assessments tools were used to assess the quality of included articles as per the NHLBI guidelines [28]. The quality assessment was conducted by one author (AKW) and then confirmed by a second author (AJB). If there was any uncertainty regarding the grade of study quality, the assessors conferred to reach an agreement. This tool contains 14 items with questions returning a ‘Yes’, ‘No’ or ‘Not Reported answer’.

## Results

### Study Selection

**Fig. 1:**
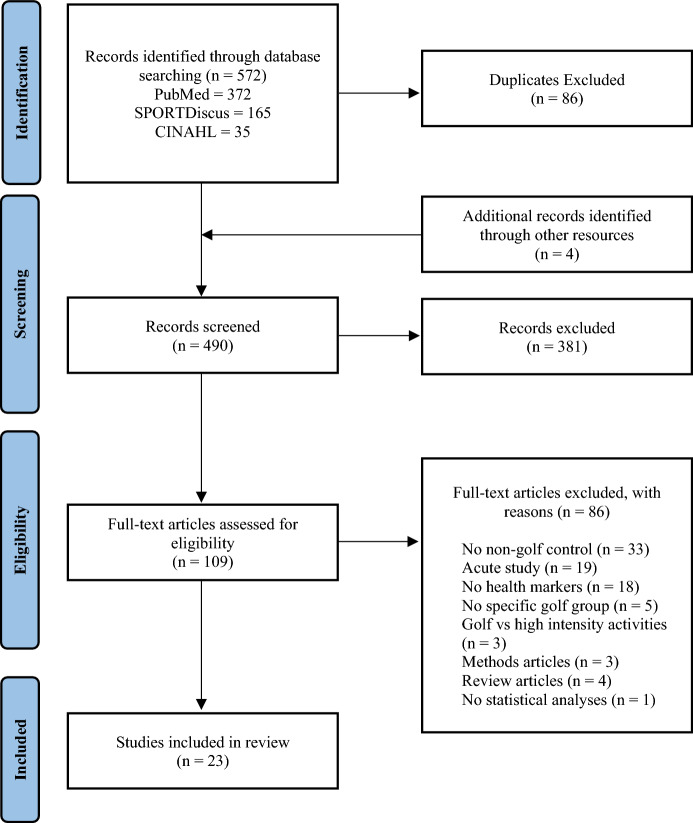
PRISMA flow diagram of the exclusion process and study results

Of the 572 articles identified, 109 full-text articles were assessed for eligibility with 23 meeting criteria and included in the full-text review (Fig. 1). Sixteen articles were rated as good quality and seven fair, and no articles were considered to have any research fatal flaws. Most articles included musculoskeletal measures (*N* = 16) along with body composition measures (*N* = 15). Fewer studies included measures of a cardiovascular nature (*N* = 5) and metabolic markers (*N* = 6), whereby the focus was predominantly on blood lipid profile. There was a total of 14 non-experimental studies and nine intervention studies (Table 1). See Table 2 for summary details of the interventions employed.

**Table 1:** Demographics, quality of studies, outcome measures and a summary of main findings of articles investigating the influence of golf activity on physical health

Study	Study design	Subject characteristics (mean ± SD)	Mode of golf club transportation	Outcome measures				Main findings	Study quality
				Musculoskeletal	Cardiovascular	Metabolic	Body composition		
Golfers									
Chang et al. [29]	Cross-sectional	Golfers: *N* = 11 females Age: 22.4 ± 2.1 y Single digit handicap 8.1 ± 6.2 h/wk 11.0 ± 2.0 y Controls: *N* = 18 Age: 22.6 ± 3.6 y	NR	Grip strength BMD	NR	NR	Fat mass Lean mass % fat	Similar grip strength, fat mass, lean mass and %fat Similar total body, hip and forearm BMD 6.7% greater lumbar spine BMD in golfers	Good
Dorado et al. [37]	Cross-sectional	Golfers: *N* = 15 Male Age: 29 ± 1 y Professional golfers for 16.7 ± 5.9 y Controls: *N* = 18 Age: 25 ± 1 y	NR	BMC Muscle mass	NR	NR	Lean mass Body fat % fat	Lean mass greater in golfers Combined arm muscle mass greater in controls but similar muscle mass in the legs No difference in BMC, body fat, % fat	Good
Du Bois et al. [30]	One Group Pre-Post	*N* = 12 males Age: 70.8 ± 4.9 y Mixed golf experience	Pulling golf clubs with pushcart. Golfers walked the course	Dynamic balance: step test Physical Performance: TUG test. 30-s chair stand Hip abductor performance: Peak force	NR	NR	NR	8.9% improvement in step time. 6.8% improvement in movement time. No change in weight shift time 13.3% improvement in up and go time. 15.9% increase in repetitions Peak and average force did not change	Good
Gao et al. [39]	Cross-sectional	Golfers: *N* = 11 Age: 66.2 ± 6.8 y Local golfers. At least 1.5 h/wk and experience 15.2 ± 13.4 y Controls: *N* = 12 Age: 71.3 ± 6.6 y	NR	Balance: Functional reach test SOT	NR	NR	NR	Golfers could reach further than controls Golfers had better scores for visual ratio and vestibular ratio but not somatosensory ratio	Fair
Herrick et al. [43]	Cross-sectional	Golfers: *N* = 31 females Age: 69.2 ± 3.5 y Non-golfers: *N* = 35 females Age: 73.4 ± 4.2 y	NR	Ultrasound Imaging: subcutaneous fat, rectus femoris, vastus intermedius, perimuscular fascia	NR	NR	BMI	% Superficial non-contractile tissue layer was significantly less in golfers (36.4) than non-golfers (41.8) Muscle thickness per kg of body mass significantly higher in golfers (0.44 cm) than non-golfers (0.30 cm) BMI significantly lower in golfers (24.8) than non-golfers (27.7)	Good
Jang et al. [38]	Cross-sectional	Screen golf frequency: ≥ 5 days/wk: *N* = 10 Age: 42.8 ± 3.3 y 2–3 days/wk: *N* = 10 Age: 43.0 ± 2.7 y 1 day/wk: *N* = 10 Age: 43.2 ± 2.7 y 0 day/wk (control): *N* = 10 Age: 43.4 ± 3.4 y Average playing duration 5.1 ± 3.5 y	NA	BMD	NR	NR	Fat mass Lean mass	Fat mass was lowest in ≥ 5 days/wk than other groups No difference in lean mass or BMD between groups	Fair
Merom et al. [44]	Cohort	Golf activity: *N* = 160 Age: 75.2 ± 4.6 y	NR	Association between golf and IRR of falls	NR	NR	NR	Unadjusted model: IRR, 0.65; 95% CI (0.47, 0.89) Adjusted model*: IRR, 0.82; 95% CI (0.57, 1.12) Adjusted model ** IRR, 1.02; 95% CI (0.73, 1.42)	Good
Müller-Riemenschneider et al. [46]	Cross-sectional	*N* = 9768 Age 45.2 ± 12.5 y; 57.3% females	NR	NR	Systolic blood pressure (SBP), diastolic blood pressure (DBP)	Triglycerides, HDL cholesterol and LDL cholesterol	BMI and waist circumference	Golf significantly associated with a higher DBP No significant association between golf and all other measures	Good
Neumayr & Lechleitner [45]	Two Group Pre-Post	*N* = 30 (53% male) Age: 54 (47–63) y Median and IQR reported	NR	NR	Resting HR_mean_ SBP_rest_ SBP_100W_ DBP_rest_ HR_100W_ HR_max_ W_max_ $\dot{V}{\text{O}}_{2\max }$ LV-Tei E/e´ ratio NT-proBNP hs TnT hs CRP CPK 24 h-HR_mean_ PVCs/h	NR	BMI Body fat %	Following golf intervention, significant reduction in resting HR, SBP and DBP. Significant reduction in HR and SBP at 100 W No significant difference in all other outcome measures	Fair
Neumayr et al. [47]	Two Group Pre-Post	*N* = 30 (53% male) Age: 54 (47–63) y Median and IQR reported	NR	NR	NR	Leucocytes hsCRP IL-6 LDL cholesterol HDL cholesterol ApoA1 TG Glucose HbA1c HOMA-IR Leptin Adiponectin FGF-21 Irisin Omentin-1 β-Trophin Resistin	BMI Body fat %	Significant increase in hsCRP, HDL-C, Irisin and Omentin-1 and significant reduction in FGF-21 following golf intervention No significant difference in all other outcome measures	Fair
Parkkari et al. [36]	Two Group Pre-Post	Golf training: *N* = 55 Age: 55 ± 4 Controls: *N* = 55 Age: 55 ± 4	Golfers walked the course	Static balance Dynamic balance Flexibility Shoulder- neck mobility Upper-body strength Trunk muscular endurance	Blood pressure HR during aerobic test $\dot{\mathrm{V}}{\mathrm{O}}_{2\mathrm{max}}$	Total cholesterol HDL cholesterol LDL cholesterol Triglycerides	BMI Waist, hip and upper-arm circumference Skin fold thickness (abdominal and triceps)	Significant reduction in BMI, waist circumference, waist-to-hip ratio, and abdominal and triceps skin fold thickness in golf training group Significantly increased trunk muscular endurance in golf training group. No difference in all other musculoskeletal performance tests No difference in $\dot{\mathrm{V}}{\mathrm{O}}_{2\mathrm{max}}$ Unchanged blood pressure in golf group. Post hoc analysis in those with highest blood pressure showed between group differences (-3 mmHg) Significantly improved submaximal aerobic performance in golf training group Significantly greater increases in HDL and ratio of HDL to total cholesterol in golf training group	Good
Podstawski et al. [31]	Two Group Pre-Post	Golfers: *N* = 25 Female 19–20 y	NR	Standing long jump 4 × 10 m shuttle run 8 s skipping and hand claps Zig-zag run Standing downward bend Barbell overhead trunk rotation 30-s sit-ups Medicine ball backward throw Medicine ball forward throw Flexed arm hang on bar 1-and 3-min Burpee test 12-min Cooper test (rowing ergometer)	NR	NR	BMI	Significant deterioration in 4 × 10 m shuttle run, zig-zag run, medicine ball forward throw, 3-min burpee test No significant change in all other motor ability tests Significant increase in BMI following golf classes	Fair
Porter et al. [48]	Cohort	Total, *N* = 13,204 Age: 54 ± 5.7 y Male, 5812; Female, 7392 Golf using a cart: *N* = 623 Golf with walking: *N* = 648	Golfers walked the course or used a cart	NR	Incident CVD (coronary heart disease, heart failure, or stroke)	NR	NR	No significant association between playing golf with a cart and golfing with walking and risk for incident CVD	Good
Schachten & Jansen [40]	Two Group Pre-Post	Golf Training: *N* = 12 Age 55.14 ± 17.64 y Social Communication: *N* = 12 Age: 53.14 ± 13.54	NA	BBS	NR	NR	NR	Significant improvements (increased) in performance within the golf training group in pre-test BBS Significant improvement in the post-test, independent of the group for BBS No significant difference between pre- and post-test BBS for either group	Good
Shimada et al. [32]	Two Group Randomised Controlled Intervention	Golf Training: *N* = 53 (male *N* = 28) Age: 70.1 ± 4.0 y Controls: N = 53 (Male *N* = 29) Age: 70.7 ± 4.7 y	NR	Walking speed Grip strength	NR	NR	NR	No significant changes observed in walking speed or grip strength	Good
Stenner et al. [49]	Cross-sectional	Golfers: *N* = 128 Age: 57.7 ± 14.2 y Non-golfers: *N* = 9307 Age: 48.5 ± 17.6	NR	NR	NR	Total cholesterol HDL cholesterol LDL cholesterol HbA1c Fasting glucose Diagnosis of diabetes, elevated blood glucose, high cholesterol, hypertension, ischemic heart disease, tachycardia, artery disease, low blood pressure, kidney disease	BMI	No difference in the odds of having high cholesterol between groups Significantly higher odds of ever having high cholesterol or being diagnosed with ischaemic heart disease for golfers. Odds increased when adjusted for physical activity. No difference when controlled for age Golfers had significantly higher odds of being overweight or obese. No difference when adjusted for age Golfers had significantly higher odds of artery disease when adjusted for physical activity No differences between groups for other measures	Good
Stockdale et al. [33]	Cross-sectional	Golfers: N = 21 Females Age: 82.9 ± 2.1 y Non-golfers: *N* = 10 females Age: 80.8 ± 1.03 y	Golfers walked the course	Relative grip strength Relative quadriceps strength TUG	NR	NR	BMI	No significant differences observed in BMI, relative grip strength and quadriceps strength between groups Golfers TUG times (10.4 ± 1.9 s) significantly faster when compared to controls (12.6 ± 3.21 s)	Good
Stroehlein et al. [50]	Two Group Randomised Control Trial	Golfers: N = 23 (female: 13; male 10) Age: 67.9 ± 4.7 y Control Group: *N* = 19 (female: 10; male 9) Age: 67.9 ± 3.9 y	NR	NR	Diabetes mellitus type 1 Diabetes mellitus type 2 Hypertension Heart disease 6-min walk test	NR	BMI	No significant difference in 6-min walk-test and cardiovascular risk factors between groups at baseline No significant difference in BMI	Good
Tsang & Hui-Chan [41]	Cross-sectional	Golfers: *N* = 11 Age: 66.2 ± 6.8 y Local golfers. At least 1.5 h/wk and experience 15.2 ± 13.4 y Elderly controls: *N* = 12 Age: 71.3 ± 6.6 y	NR	Stability tests: Reaction time maximum excursion directional control Knee joint repositioning test	NR	NR	NR	Golfers had significantly better knee joint proprioceptive activity and less angle errors in the knee joint repositioning test compared to elderly controls Golfers achieved significantly faster reaction times, better maximum excursion scores and directional control compared to elderly controls (Reaction time: Golfers: 0.8 ± 0.2 s, Elderly controls: 1.0 ± 0.3 s) (Maximum excursion: Golfers: 92.9 ± 5.7%, Elderly controls: 83.2 ± 8.2%) (Directional control: Golfers: 78.3 ± 5.4%, Elderly controls: 70.3 ± 7.3%)	Fair
Tsang & Hui-Chan [42]	Cross-sectional	Golfers: N = 11 males Age: 66.2 ± 6.8 y Controls: N = 12 males Age: 71.3 ± 6.6 y	NR	Balance Tests: Single-leg stance (s), perturbed single-leg stance (centre of pressure excursion angle), forward-lunge (distance/body height)	NR	NR	NR	Significantly longer single-leg stance duration in golfers (28.1 s) than controls (17.1 s) Significantly smaller sway angles in anterior and posterior direction in golfers Significantly larger normalized lunge distances in golfers (dom = 54.1%, non-dom = 53.8%) than controls (dom = 46.0%, non-dom = 46.7%)	Good
Webb et al. [51]	Cross-sectional	Golfers: *N* = 21 females Age: 83 ± 3 y Non-golfers: *N* = 10 females Age: 80.5 ± 1.3 y	NR	NR	NR	NR	BMI	No significant difference observed in BMI between groups	Good
Caddies									
Goto et al. [34]	Two-group Longitudinal	Caddies: *N* = 6 females Age: 37.8 ± 2.6 y Controls (desk workers): *N* = 6 Females Age: 40.8 ± 4.9 y	Caddies walked the course	BMD: lumbar spine total, proximal femur Leg press strength Grip strength	NR	Bone metabolic markers: alkaline phosphatase, urine deoxypyrisnoline, urine pyrisinoline, vitamin D_3_, parathyroid hormone	BMI	Significant improvements in lumbar (+ 0.009 g/cm^2^) and proximal femur (+ 0.022 g/cm^2^) BMD in caddies Significant increases in leg press strength in caddies (18%) No significant trends identified in bone metabolic markers No significant differences in BMI between groups, or across time points	Good
Hoshino et al. [35]	Cross-sectional	Caddies: *N* = 74 Females Controls: *N* = 433 Females Participants were age matched (range: 20–59 y) and split by age category (20–39, 40–49, 50–59 y) and menopausal status	NR	Ultrasound Densitometry: Achilles stiffness index Quadriceps muscle strength (age group 45–59) Grip strength (age group 45–59)	NR	Urinary pyridinoline and deoxypyridinoline levels	BMI	Significantly greater stiffness index (%) in caddies than controls for all three age categories (20–39, 40–49, 50–59 y) and in pre- and post-menopausal status Significantly greater quadriceps strength and grip strength in caddies than controls No significant increases in pyridinoline and deoxypyridinoline levels in caddie group No significant difference in BMI between caddies and controls for all three age categories	Fair

*24 h-HRmean* mean heart rate by 24-h ECG, *ApoA1* apolipoprotein A1, *BBS* Berg balance scale, *BMC* bone mineral content, *BMD* bone mineral density, *BMI* body mass index, *CPK* creatine phosphokinase, *CVD* cardiovascular disease, *DBP* diastolic blood pressure, *E/e’* ratio of early mitral inflow velocity-to-early diastolic tissue velocity, *FGF-21* fibroblast growth factor-21, *HbA1c* glycated haemoglobin, *HDL* high-density lipoprotein, *HOMA-IR* homeostatic model assessment for insulin resistance, *HR* heart rate, *HR*_*100W*_ heart rate at 100 watts, *HR*_*max*_ maximum heart rate, *hs CRP* high-sensitivity C-reactive protein, *hs TnT* high-sensitivity troponin T, *IL-6* interlukin-6, *IQR* interquartile range, *IRR* incidence rate ratios, *LDL* low-density lipoprotein, *LV-Tei* left ventricular Tei Index, *N* number of participants, *NR* not reported, *NA* not applicable, *NT-proBNP* N-terminal-pro B-type natriuretic peptide, *PVCs/h* premature ventricular contractions per hour, *SBP* systolic blood pressure, *SBP*_*100W*_ systolic blood pressure at 100 watts, *SD* standard deviation, *SOT* sensory organisation test, *TUG* timed up and go, $\dot{V}{\text{O}}_{2\max }$ maximum oxygen uptake, *W*_*max*_ maximum power output

^*^Model adjusted for age, light-intensity sport score, moderate-intensity sport score, strenuous-intensity sport score, muscle strengthening score, walking score, and lifestyle physical activity score. Lifestyle physical activity score includes functional activities, such as light housework, heavy housework, lawn and yard care, caregiving, and paid or volunteer work

^**^Model adjusted for ‘*’ in addition to risk factors for falls

**Table 2:** Summary of intervention study information relating to golf and health

Study	Intervention	Frequency	Adherence
Du Bois et al. [30]	12-week comprehensive golf training programme	Twice weekly for 90 min per session	Average of 91%. Attended a minimum of 75% of the sessions
Goto et al. [34]	12 months of caddying	An average of 20,499 steps when caddying	Daily record of pedometry with 100% reporting for a period of 12 months
Neumayr and Lechleitner [45]	1-week intervention of golf course play	33.5 h per week Average of 4.8 h per day	Not reported. Participants were free to take a day off during the intervention week
Neumayr et al. [47]	1-week intervention of golf course play	33.5 h per week Average of 4.8 h per day	Not reported. Participants were free to take a day off during the intervention week
Parkkari et al. [36]	20-week intervention of golf course play	Golf Group: 18-hole round of golf twice a week. All participants walked the golf course Control Group: Continued sedentary lifestyle. Continued summer activities such as gardening and home repair	Average of 2.5 ± 1.1 rounds of golf per week (10 ± 4 h per week)
Podstawski et al. [31]	Golf classes across a university semester (5 months)	90-min golf classes × 15 (minimum)	Every student had to attend 15, 90-min classes
Schachten et al. [40]	10-week intervention consisting of golf training programme	Golf Group: Golf training twice a week for 1 h, which resulted in 20 sessions Control group: Social communication meeting including group discussion, reading, and common game activities	Each participant attended at least 18 of the 20 training sessions
Shimada et al. [32]	24-week intervention consisting of golf sessions	Golf group: 14 practice sessions and 10 golf course sessions (duration: 90–120 min each) Control group: Two 90-min health education classes focused on health promotion during the study period	Golf programme 96.2% (51/53 participants)
Stroehlein et al. [50]	22-week intervention consisting of golf training	Golf group: Golf training consisted of 3 × sessions per week. Sessions lasted 60 min 18 practice sessions and 25 sessions at the driving range Control group: Asked to keep their lifestyle and sports activities unchanged	Overall adherence to the golf training was 75%, which can be considered as acceptable

### Musculoskeletal Effects

An overview of results is shown in Table 1. Sixteen studies included measures of a musculoskeletal nature, including strength and endurance measures [29–36], BMD or BMC [29, 34, 37, 38], balance and stability measures [30, 36, 39–42], muscle mass and thickness [35, 37, 43], flexibility and mobility [36], or physical competency measures [30, 32, 33, 44]. Musculoskeletal strength and endurance results were mixed, particularly amongst cross-sectional studies, where Stockdale et al. [33] observed no statistically significant differences in grip (golfers: 0.33 ± 0.06 N/kg; non-golfers: 0.29 ± 0.06 N/kg) or quadriceps strength between golfers and non-golfers (golfers: 2.78 ± 0.74 N/kg; non-golfers: 2.69 ± 0.83 N/kg). Chang et al. [29] also identified no significant difference in grip strength in golfers in comparison to control subjects. Mixed results were observed for intervention studies, whereby no significant changes were observed in grip strength in age-matched participants (golf mean difference = 0.85 kg, 95% confidence interval (CI): 0.04–1.67); control mean difference = 1.26 kg, 95% CI 0.40–2.13) [32]. Positive changes were, however, noted in one golf-training study where static back-extension time increased in the golf training group (from 93 to 101 s), whereas no change was observed for controls (91–89 s) [36].

BMD results were mostly non-significant; Chang et al. [29] noted 6.7% greater lumbar BMD in golfers than control—however, total body and hip BMD were similar. Other studies with golfers found no significant differences in BMD [37, 38]. Stability and balance measures were largely positive for golfing groups, whereby improvements were shown after training (8.9% improvement on step test [30]) and when analysed cross-sectionally in comparison to a control population [39, 41, 42]. Despite a low number of papers with muscle mass and thickness measures, results were largely positive, with increased muscle mass noted in the arms [37] and increased muscle thickness in golfers in comparison to non-golfers [43]. Mixed results were shown for physical competency measures, where no change was observed in walking speed after a golf programme [32]; however, improvements in timed up-and-go (TUG) tests were observed [33] or were greater than in non-golfers (13.3%) [30].

Studies in caddies mostly showed positive results, with Achilles tendon stiffness index greater in caddies across three age groups in comparison to control participants [35]. Additionally, caddies demonstrated increased quadriceps strength [34, 35], with improvements in BMD also observed [34].

### Cardiovascular Effects

Five studies considered cardiovascular measures (Table 1), including DBP and SBP [36, 45, 46], resting heart rate and aerobic performance [36, 47], and risk of cardiovascular disease [48, 49]. In the large cohort study conducted by Müller-Riemenschneider et al. [46], golf was associated with increased DBP in both models evaluated (Model 1: Effect size = 2.04, 95% CI 0.44–3.64; Model 2: Effect size = 1.85, 95% CI 0.28–3.42). In other studies where SBP and DBP reduced after a golf intervention [45], it should be noted that the quality of the study was only fair, or there was a trend towards reducing a high DBP only when subjects with a pre-intervention high DBP were considered (between group differences of – 3 mmHg in favour of golf intervention) [36]. Both studies that observed a cardiovascular disease found golf was not associated with disease incidence [48, 49]. It is, however, to be noted that the cross-sectional study only made this comparison as a single time point, and thus cannot determine causality [49]. In addition, aerobic performance determined through $\dot{V}{\text{O}}_{2\max }$ [45] or 6-min walking test did not differ between golfers and controls [50], while submaximal exercise performance improved after golf training with lower SBP, HR at 100 W [45], and lower oxygen consumption, HR and lactate at 7 METs [36].

### Metabolic Effects

Six studies included measures of a metabolic nature (Table 1). This included measures of blood lipid profile (total, high-density lipoprotein cholesterol [HDL], low-density lipoprotein cholesterol [LDL] and triglycerides) [36, 46, 47, 49] or within a caddy population, measures of bone metabolic markers [34] or bone resorption rates through urinary pyridinoline and deoxypyridinoline levels [35]. Mixed results were observed for blood lipid profiles, including no significant associations with golf [46], significantly higher odds of ever having high cholesterol – although not when controlled for age [49], positive results for HDL levels (training effect-adjusted mean difference between groups 0.05 mmol/L (95% CI 0.00–0.10); HDL-C improvement of 5 mg/dL) and the ratio of HDL to total cholesterol (training effect-adjusted mean difference between groups 1.2% (95% CI 0.2–2.2)) [36, 47]. Urinary pyridinoline and deoxypyridinoline levels were found to not increase in caddies in comparison to controls pre- and post-menopause [35]. However, no significant trends were identified in bone metabolic markers [34].

### Body Composition Effects

BMI data were included in 12 studies (Table 1). Mixed results were observed with no differences in BMI when considered cross-sectionally [33, 35, 39, 46, 49, 51], with only Herrick et al. [43] noting lower BMI in golfers (24.8 ± 2.5 kg·m^−2^) in comparison to controls (27.9 ± 3.5 kg·m^−2^). Longitudinal studies showed similar outcomes for BMI measurements, with significant reductions noted in fewer studies [36] than those that had no change or differences between groups [33, 34, 45, 47]. Studies that measured lean or fat mass largely found positive results, including a − 8% change in abdominal skin fold measurement [36, 37] rather than no change [29].

## Discussion

This systematic review investigated the effects that golf participation has on physical health, specifically musculoskeletal, cardiovascular and metabolic health and body composition. Although a previous scoping review has outlined the physical health benefits that golf can provide for an individual, further research was required to systematically describe the relationship between golf participation and physical health [18]. To our knowledge, this is the first systematic review addressing the impact that golf participation, including golf play and caddying, has on cardiovascular, metabolic and musculoskeletal health, as well as body composition. Overall, the review highlighted that golf play can, in some instances, positively impact measures of physical health; however, findings across domains were mixed. Furthermore, when considering the population of golf caddies, findings highlighted that caddying may positively affect musculoskeletal health, although research remains limited in this population.

### Musculoskeletal Effects

#### Muscular Strength and Size

Golf may be beneficial for the preservation of muscle mass and thickness. However, it must be noted that findings conflicted regarding the lower limbs, with one article observing similar muscle mass in young male golfers (29 ± 1 years) and controls [37]. In contrast, a later study demonstrated that older female golfers (69 ± 4 years) had larger relative quadriceps muscle thickness than non-golfers [43]. The latter observation is of particular importance, since age is associated with sex-independent reductions in the muscle/body weight ratio [52]. Sarcopenia, an age-related loss of skeletal muscle mass and strength [53], has been demonstrated through meta-analytical study to be associated with increased falls and fractures in older adults [54].

While hormonal changes are in part responsible for sarcopenia, environmental declines in PA are also contributory [53]. Golf offers the possibility for increasing PA of a light-moderate intensity [26], which may contribute towards the PA guidelines published by the WHO [55]. Thus, golf participation can be encouraged in order to achieve PA recommendations [26]. It is possible that with increasing PA that is likely to occur with on-course golf activity [56], golf may provide a stimulus for lower limb muscular hypertrophy. While this proposal relates to muscle thickness from limited research, the potential benefits of golf did not extend to muscular strength. From the four studies that investigated muscular strength in golfers, the consensus was that there was similar grip strength between golfers and non-golfers. Similarly, specific to lower body muscular strength, only two studies have been conducted and both found that golf was not beneficial for either quadriceps strength [33] or peak hip abductor force [30]. Golfers were reported to have walked the course, with one study describing that clubs were transported using a pushcart [30], which was outlined within the original methodological protocol article [57]. Although the METs for walking and pulling clubs with a cart (5.3 METs) are greater than walking while carrying clubs (4.3 METs) [23], the latter mode may require an additional effort. Indeed, caddies can be expected to carry a bag of at least 12.5 kg [3], and this may, in part, explain contrasting observations in the golfing literature, since hand grip and quadriceps strengths were greater in caddies than in non-caddies [34, 35]. Through cross-sectional observations, Hoshino et al. [35] reported in a small sample of long-term golf caddies who carry clubs that quadriceps strength was greater in caddies by 18.1 kg (difference in means) than in controls. The consequential benefits that may arise from carrying an additive load rather than solely walking are promising, and are likely to also contribute towards the PA guidelines, with the inclusion of strength-based exercise on at least 2 days per week [17, 55].

Additionally, drawing comparisons between golfers and caddies is challenging as the conflicting findings may be due, but not limited to, the differences in the transportation of clubs, the volume of activity completed, and/or the participant demographics. Indeed, the exercise stimulus may not have been sufficient in the longitudinal study [30] and the older aged population used by Stockdale et al. [33] (see Tables 1 and 2 for descriptions). Moreover, previous research has indicated that the step count completed by golfers was 11,948 ± 1,781 per round [58], while caddies have been reported to complete 20,499 ± 812 per round [34]. While the variation in activity is noteworthy, it is of course important to note that the golf course and skill level would influence this.

#### Balance

Most studies that investigated balance demonstrated a positive influence from golf. Proactive balance tests, described as anticipation of predicted disturbances [59], including functional reach, were greater in golfers [39], and the TUG test was faster in golfers than in non-golfers [33], improving by 13.3% after 12 weeks of golf practice [30]. As noted by Stockdale et al. [33], physical performance from the TUG test indicated that non-golfers were below the threshold and consequently classed as sarcopenic, whereas the golfers exceeded this threshold for the prediction of sarcopenia. As a test of functional ability [60], the TUG test is an important predictor of falls in seniors [61], and the potential benefits from golf for superior functional ability are welcomed. Other performance tests, such as the Berg Balance Scale (BBS), considered the gold-standard for balance assessments [59, 62], indicated further benefits of golf. Schachten and Jansen [40] found that 10 weeks of golf training improved BBS in middle-aged stroke survivors. Still, similar improvements were also noted for the parallel social communications group, although this study lacked randomisation into each treatment arm. In contrast, a two-group pre-test post-test golf intervention for 20 weeks suggested static and dynamic balance did not differ [36]. Thus, more intervention studies are required with adequate randomisation to determine the influence of golf on functional balance tests. One study reported a significantly lower incidence rate ratio (IRR) of falls for golfers compared with other leisure-time physical activities (LTPAs) when PA of multiple intensities was considered [44]. Although this association was dampened after controlling for LTPA and history of falls, this may suggest that a prominent element of golf (i.e., PA) is an important and mediating factor in the risk of falls. This information is of significant clinical relevance, since falls are the second leading cause of unintentional mortality [63]. The number of deaths from falls within England during 2019 increased with age and totalled 6,138 in those > 40 years of age [64]. The ramifications for golf to aid in the preservation of muscular performance and balance are promising, with the intention of maintaining functional ability and, by extension, healthy ageing [65].

#### Bone Mineral Density

Studies identified within the review highlight that playing golf has minimal effects on total body BMD or BMC [29, 37, 38]. The influence of regional BMD is contradictory, however, with Chang et al. [29] reporting 6.7% greater lumbar spine BMD in female elite golfers compared to controls. However, Jang et al. [38] and Dorado et al. [37] reported no differences in spine BMD when comparing male golfers participating in screen golf against control subjects. It should be noted that screen golf does not take into consideration walking on the golf course and the transportation of golf clubs; therefore, this may explain the conflicting findings. However, the reporting of transportation of clubs in other studies was lacking, thus it is not clear at present whether walking a course whilst carrying is influential. Sex differences may also be contributory, since the rate of production and loss in BMD differs between women and men [66]. This may help to provide some insight as to why Chang et al. [29] observed greater spine BMD in golfers, while male golfers [38] of a similar and younger age were comparable to controls.

In consideration of golf caddies, positive findings were observed in relation to BMD and Achilles stiffness index [34, 35]. Specifically, Goto and colleagues [34] reported that female golf caddies, at a pre-menopausal stage, significantly increased lumbar spine BMD at 6 and 12 months and proximal femur BMD following 12 months of caddying. When taking into consideration the activity levels of these two groups, caddies walked over threefold the distance of the desk workers. Moreover, since oestrogen deficiency plays a key role in a net loss of bone [67], bone production in the pre-menopausal state may be important for the preservation of BMD, facilitated by caddying.

### Cardiovascular Effects

From the limited literature exploring the influence of golf on blood pressure, the findings may be conditional on both the training intervention and baseline blood pressure. Two studies observed reductions in SBP and DBP [45, 47], which represent beneficial changes since high blood pressure is a modifiable risk factor for CVD [68]. However, the participants completed the golf training as a vacation, and thus stress may confound the association between golf training and markers of health, as suggested by the authors, concomitant with reduced fibroblast growth factor 21 (FGF-21) [47]. In contrast, a longer intervention of 20 weeks, which may better represent the influence of sustained golf play, suggested that blood pressure did not change based on group data [36]. However, subgroup analysis in those with the highest blood pressures observed -3 mmHg in DBP in the golf group compared to controls. While this observation is not supported by cross-sectional research having shown a positive association between golf and DBP [46], it may support the proposition through meta-analysis that those with hypertension may benefit the most from endurance training [69]. Moreover, the long-term impact of golf on CVD risk requires further substantiation, since one article identified no significant association with CVD incidence [48]. Additional work, whilst not included in the review due to a lack of a no-golf comparator/reference group, suggests that mortality is approximately 60% compared to that of the estimated general population [70]. Thus, future work is required to determine the long-term health implications and associated risk for CVD following golf participation.

Cardiorespiratory fitness ($\dot{V}{\text{O}}_{2\max }$) is a strong predictor of mortality in men and women [71]. However, $\dot{V}{\text{O}}_{2\max }$ did not change after 1 week [45] or 20 weeks [36] of golf training, and nor did maximum power output (*W*_max_) [45]. It is possible that the intensity of golf was not sufficient to induce central adaptations in left ventricular function [45] and/or peripheral adaptation to elicit changes in aerobic fitness. However, low volume, high-intensity interval exercise in older men has yielded benefits in $\dot{V}{\text{O}}_{2\max }$ [72] and peak power output [73]. Nonetheless, studies in this review did observe improvements in submaximal exercise performance [36, 45] and, thus, this suggests less cardiovascular demand with superior exercise economy after the golf training, despite no changes in $\dot{V}{\text{O}}_{2\max }$.

### Metabolic Effects

Several metabolic variables were investigated (Table 1) but with mixed findings, making it challenging to propose a consensus concerning metabolic health parameters. However, more frequently investigated was the blood lipid profile, with reports of no association with golf and triglycerides, HDL cholesterol and LDL cholesterol [46]. Intervention studies, however, reported increased HDL-C [47] and the ratio of HDL to total cholesterol [36] following golf training. This is encouraging since HDL is an important predictor of CVD risk and higher HDL is inversely associated with coronary heart disease [74, 75]. However, although cross-sectional analysis golfers were at greater odds of being diagnosed with high cholesterol than non-golfers [49], this difference was abolished when controlling for both age and physical activity, which may be stronger determinants of total cholesterol than golf participation.

The disparity in results relating to blood lipid profile may be due in part to study designs. Both intervention studies, irrespective of duration, provided positive results after golf training, whereas the cross-sectional studies, which are likely influenced by confounding between-subject variables, were negligible or negative. Thus, additional confirmatory studies concerning long-term golf play and metabolic health are required.

### Body Composition Effects

Most cross-sectional [33, 39, 46, 49, 51] and intervention [45, 47] studies indicated that playing golf or caddying [34, 35] does not impact BMI. Some studies, however, have suggested the contrary, that BMI was lower in golfers than controls [43], reduced following a 20-week golf season [36], or increased by 0.7% following at least 15 × 90-min golf classes over a 5-month period [31]. Nevertheless, the true physiological significance of a 0.17 kg^.^m^2^ increase as reported in the latter warrants mention. These studies observing changes in BMI were in the minority, but the contradiction may arise from the methodological inconsistencies of the golf interventions and participant demographics, making it difficult to draw sound conclusions in relation to the impact of within-subject long-term golf play and BMI. Indeed, BMI is associated with all-cause mortality in a J-shaped relationship [76], and an apparent lack of evidence to suggest golf produces a universal change in BMI, which could be inferred as both beneficial and deleterious depending on baseline BMI.

BMI may not be suitable for differentiating fat mass from lean mass, especially when assessing BMI change [77]. The influence of golf on body composition from cross-sectional studies was equally undecided as to whether golf is beneficial [29, 37, 38, 46]. From intervention studies, fat mass did not differ between golfers and controls in two intervention studies; however, they were only 1 week in duration [45, 47]. In contrast, a more substantial intervention period of 20 weeks yielded reductions in abdominal skinfold thickness and waist circumference [36]. These observations are of particular importance since central adiposity/waist circumference is a key component of the metabolic syndrome [78], which represents a constellation of risk factors associated with greater risk for developing CVD and type 2 diabetes mellitus [79]. Before a stronger conclusion can be elucidated, however, more intervention studies that are of sufficient duration are necessary.

### Strengths and Limitations

The current review provides valuable insight into the health benefits of playing golf and caddying; however, there are some noteworthy limitations to consider. Whilst efforts were made to ensure a rigorous and thorough search process, it is possible that some articles may have been missed, which may include peer-reviewed journal articles not written in English. The reviewed studies provided a variety of study designs and wide heterogeneity. Additionally, the golfers and caddies differed in sex, age and sample size, and, as a result, a meta-analysis was not feasible at this moment in time. The review was limited to four outcome-measure categories in relation to health; therefore, there may be other outcome measures relevant to the health benefits of golf and caddying. However, the intention was to focus on physical health; other studies should continue to establish the influence of golf on mental health and wellbeing.

The NHLBI risk assessments tools demonstrated that the quality of included studies was ‘fair’ to ‘good’. In relation to intervention studies, approximately 70% were rated ‘good’, suggesting the current evidence is strong. While many longitudinal studies were rated ‘good’, these studies varied considerably with regard to: heterogeneity of participants, duration of the intervention (1 week to 12 months), golf activity performed, and frequency of activity. It is, therefore, difficult to draw sound recommendations regarding duration, activity and frequency of golf interventions to improve health. A limited number of studies (*N* = 2) investigated the health benefits of caddying; nevertheless, the initial evidence suggests that golf caddying may have a positive impact on musculoskeletal health. To draw stronger conclusions within this area, however, additional longitudinal research is required, particularly within the male population. Furthermore, a limited number of studies (*N* = 5) reported specifically that golfers or caddies walked the course [30, 33, 34, 36, 48], and only two studies indicated the golfers’ club transportation method [30, 48, 57]. As previously discussed, the metabolic demand of golf varies depending on the golfer’s club transportation choice and if they walk the course or use a motorized golf cart [23, 26]. Therefore, future golf course participation-related studies should take into consideration golfers’ and caddies’ mode of transportation (e.g., walking or using a motorised cart) and club transportation (e.g., carrying clubs, pulling or pushing a cart) on the golf course, when initially recruiting their sample. In addition, this information should be reported within studies to improve clarity, which will enable appropriate between-study key findings. Moreover, whilst beyond the direct scope of this review, interest has increased relating to the adverse effects of golf participation pertaining to injuries and musculoskeletal and cardiovascular risks, in addition to skin-related issues associated with golf participation [3, 18, 80, 81]. Accordingly, more studies are required to fully understand the depth and breadth of the impact that golf has on both players and caddies [3].

Beyond the limitations, this review is the first to systematically collate the literature in relation to the health benefits of playing golf and caddying. Thus, this review advances our understanding of the potential impact that playing golf and caddying can have on body composition and musculoskeletal, cardiovascular and metabolic health.

### Conclusions

Golf may be an effective method for improving musculoskeletal, cardiovascular and metabolic health. Additionally, most of the evidence suggests that playing golf does not influence body composition (BMI). Yet, there was also evidence to suggest that golf was not beneficial for the domains studied, although this may be dependent on: the sample of golfers, study design, the length of the intervention, and the frequency of activity. Consequently, the influence of playing golf on physical health requires further study with consideration of such methodological factors. Furthermore, the initial evidence suggests that golf caddying may positively impact musculoskeletal health; however, it would be appropriate to conduct further investigations within this area due to the limited literature at present.

## Declarations

### Data availability

There are no underlying data to share.
